# Determinants of the Number of Tetanus‐Toxoid Injections Before Birth Among Pregnant Women in Somalia: A Multilevel Analysis

**DOI:** 10.1002/puh2.70355

**Published:** 2026-09-10

**Authors:** Jama Mohamed, Hamse Mohamed Amoud, Ahmed Ismail Mohamed, Abdisalam Hassan Muse, Azizur Rahman, Saralees Nadarajah

**Affiliations:** ^1^ College of Business, Economics and Statistics Faculty of Statistics and Data Science University of Hargeisa Hargeisa Somalia; ^2^ School of Postgraduate Studies Amoud University Borama Somalia; ^3^ College of Medicine and Health Sciences, Faculty of Nutrition and Food Science University of Hargeisa Hargeisa Somalia; ^4^ Research and Innovation Center Amoud University Borama Somalia; ^5^ School of Computing, Mathematics and Engineering Charles Sturt University Wagga Wagga New South Wales Australia; ^6^ Department of Mathematics University of Manchester Manchester UK

**Keywords:** antenatal care, maternal immunization, mixed‐effects negative binomial model, neonatal tetanus, Somalia, vaccine hesitancy

## Abstract

**Background:**

Maternal and neonatal tetanus remains a major public health concern in Somalia, where low immunization coverage contributes to preventable mortality. Despite global progress, Somalia continues to report suboptimal tetanus‐toxoid (TT) vaccine uptake due to conflict, weak health systems, and socioeconomic barriers. This study examined multilevel factors associated with the number of TT injections before birth among Somali women.

**Methods:**

We analyzed data from the Somali Health and Demographic Survey (2020), including 4841 pregnant women aged 15–49. A mixed‐effects negative binomial regression model addressed overdispersion, excess zeros, and hierarchical structure (region and residence). Model fit was assessed using Akaike (AIC) and Bayesian (BIC) information criteria.

**Results:**

Only 32.6% of women received at least one TT injection, whereas 67.4% received none. Higher TT uptake was associated with older age (IRR = 1.62; 95% CI = 1.09–2.42), higher wealth (IRR = 1.49; 95% CI = 1.01–2.18), prior vaccination (IRR = 1.76; 95% CI = 1.44–2.16), antenatal care visits (IRR = 1.02; 95% CI = 1.01–1.04), employment (IRR = 1.41; 95% CI = 1.00–1.97), clinic deliveries (IRR = 1.23; 95% CI = 1.01–1.50), and health insurance (IRR = 5.24; 95% CI = 1.04–26.47). Conversely, zero uptake was more likely among nomadic women (AOR = 1.40; 95% CI = 1.06–1.85), those with no prior vaccination (AOR = 3.64; 95% CI = 2.76–4.80), home deliveries (AOR = 1.42; 95% CI = 1.18–1.71), no phone ownership (AOR = 1.31; 95% CI = 1.02–1.68), and women whose husbands lacked education (AOR = 1.27; 95% CI = 1.02–1.58).

**Conclusion:**

TT vaccine coverage among pregnant women in Somalia remains critically low and is shaped by socioeconomic inequities, healthcare access, and household decision‐making. Strengthening antenatal care integration, targeted outreach for nomadic populations, economic empowerment, health education, and expanding insurance coverage are essential to improve maternal immunization and eliminate neonatal tetanus.

## Graphical abstract

Using nationally representative data from Somalia, this study reveals substantial gaps in maternal tetanus vaccination coverage and identifies key determinants of vaccine uptake, including socioeconomic status, healthcare access, and antenatal care utilization. Targeted interventions are needed to accelerate maternal and neonatal tetanus elimination.

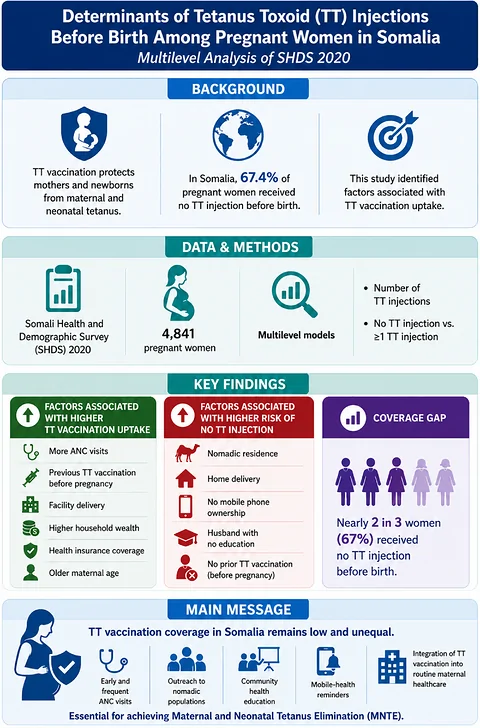

## 1 Background

Tetanus is a serious bacterial infection caused by *Clostridium tetani*, which releases a neurotoxin that induces severe muscle contractions and can be fatal. It poses a greater risk and is more severe among neonates and pregnant women who have not received adequate immunization with tetanus‐toxoid (TT)‐containing vaccines, particularly in areas with poor hygiene [1, 2]. It remains a major contributor to neonatal and maternal mortality across many sub‐Saharan African countries [1]. One of the most effective ways to prevent maternal and neonatal tetanus (MNT) is through the TT vaccine [3]. The World Health Organization (WHO) also recommends this vaccine as the primary preventive measure for pregnant women to protect the mother and newborn from neonatal tetanus [4]. Countries should give special attention to women at reproductive age to ensure they are immunized in regions where MNT remains a public health concern and where maternal and neonatal tetanus elimination (MNTE) has not yet been achieved [4]. Completing the full five‐dose TT vaccination schedule provides long‐term immunity, offering protection throughout the reproductive years [5]. TT vaccination during pregnancy is crucial in preventing neonatal tetanus, reducing associated mortality by up to 94% [6, 7]. In conflict‐affected areas with limited access to healthcare, neonatal mortality rates range from 80% to 100% [8, 9, 10].

Despite global progress in maternal and neonatal healthcare, tetanus remains a significant public health concern, particularly in low‐resource settings. As of December 2024, efforts toward global MNTE have led to 49 out of 59 at‐risk countries achieving elimination since 2000, whereas 10 countries continue to face MNT as a public health issue; notably, in 1999, 57 countries had not yet reached elimination status, underscoring the continued need for targeted interventions [11]. Globally, an estimated 24,000 newborns died from neonatal tetanus in 2021, representing an approximately 88% reduction from an estimated 200,000 neonatal deaths in 2000, reflecting the substantial impact of global maternal immunization and MNTE initiatives [11]. These remaining countries still carry a high burden of neonatal tetanus due to restricted access to maternal immunization programs, inadequate healthcare infrastructure, and disparities in antenatal care services [12]. The Global MNTE Project has significantly reduced cases through widespread immunization campaigns; however, persistent challenges, such as limited healthcare access, socioeconomic barriers, and vaccine hesitancy, continue to hinder progress [13]. Globally, 75% of expectant mothers are currently immunized with two or more doses of the TT vaccine (TT2+). However, coverage varies substantially—from 95% in Southeast Asia to 63% in Africa and as low as 53% in the Eastern Mediterranean region [14].

Studies conducted in Africa indicate that neonatal tetanus remains a leading cause of newborn mortality, particularly in countries with low immunization coverage, although the factors influencing immunization rates vary across nations [15]. In Kenya, adequate tetanus immunization is associated with lower birth order, higher household wealth, women's employment, joint health decision‐making, and increased antenatal care visits [16]. In contrast, Ethiopia faces persistently high maternal and newborn tetanus incidence due to a large unimmunized population, weak health systems, poor reporting, limited resources, lack of healthcare facilities, insufficient professional guidance, and fear of vaccine side effects [17]. The highest maternal and newborn tetanus rates in Ethiopia were primarily driven by inadequate antenatal care and pronounced socioeconomic disparities [17]. In Nigeria, up to 66% of women experience missed opportunities for tetanus vaccination, further contributing to low coverage [16]. Uganda shows relatively higher coverage, with most women receiving at least two doses of the TT vaccine; however, significant disparities persist, particularly among rural, younger, and less‐educated women, and coverage varies across regions and income levels [18]. Across East Africa, more broadly, only 41% of expectant mothers receive the recommended two or more doses of the TT vaccine, highlighting the urgent need to improve healthcare practices and expand access to immunization services [15].

Somalia remains among the countries that have not yet achieved MNTE status and continues to be classified as a country where MNT remains a public health concern [11]. Reliable national estimates of MNT cases and deaths are unavailable because surveillance and case reporting remain limited, resulting in substantial underreporting of cases [11, 19]. Although Somalia has made progress through immunization initiatives and support from international partners, neonatal tetanus remains a preventable cause of morbidity and mortality because of persistent gaps in maternal protection. The continued risk of MNT in Somalia is associated with inadequate maternal tetanus‐containing vaccine coverage, limited antenatal care utilization, low rates of skilled birth attendance, suboptimal delivery and cord‐care practices, and challenges in reaching populations living in remote and nomadic settings [20]. Achieving adequate protection against neonatal tetanus in these settings requires women to receive the recommended number of tetanus‐containing vaccine doses according to their previous vaccination history. In Somalia, prolonged conflict, population displacement, weak health infrastructure, and limited access to essential maternal health services have affected the delivery and utilization of preventive healthcare interventions, including maternal immunization [20]. Therefore, improving maternal tetanus vaccination coverage requires addressing not only vaccine availability but also socioeconomic, healthcare access, and community‐level barriers influencing vaccine uptake.

The Somali immunization program operates within the framework of the Expanded Programme on Immunization (EPI), led by the Federal Ministry of Health in collaboration with international partners, including the WHO, UNICEF, and Gavi, the Vaccine Alliance [21, 22]. Since its establishment, the EPI has served as the primary platform for delivering essential vaccination services in Somalia through routine immunization and supplementary immunization activities, with continued efforts to strengthen vaccine supply systems, cold‐chain infrastructure, healthcare worker capacity, and access to immunization services, particularly in conflict‐affected and hard‐to‐reach populations [21, 23]. Maternal tetanus vaccination is integrated into routine maternal health services, particularly ANC, where pregnant women are assessed according to their previous tetanus‐containing vaccine history and receive TT‐containing vaccines when eligible. This strategy aligns with the global MNTE framework, which promotes vaccination of women during pregnancy through ANC platforms to provide protection against MNT [24]. Nevertheless, coverage remains inadequate. According to the Somali Health and Demographic Survey (SHDS) 2020, a substantial proportion of women did not receive tetanus vaccination during pregnancy, demonstrating persistent gaps in maternal immunization services [25]. These challenges reflect broader health‐system constraints, including limited healthcare availability, unequal access between urban and rural populations, and difficulties reaching underserved communities [20].

Evidence from low‐ and middle‐income countries indicates that maternal tetanus vaccination uptake is influenced by multiple individual and contextual factors, including maternal education, household socioeconomic status, place of residence, exposure to health information, ANC utilization, and availability of healthcare services [26]. However, evidence regarding the determinants of the number of tetanus‐containing vaccine injections received among pregnant women in Somalia remains limited. Previous studies in Somalia have largely focused on general immunization coverage [27], maternal health service utilization [28], or childhood vaccination [29], whereas the factors associated with completion of maternal tetanus vaccination schedules have received limited attention.

Therefore, this study used nationally representative data from the SHDS 2020 to identify individual‐ and community‐level factors associated with the number of TT injections received before birth among pregnant women in Somalia using a multilevel analytical approach. Understanding these determinants is essential for designing targeted interventions to improve maternal immunization coverage, strengthen antenatal care integration, and support Somalia's efforts toward achieving MNTE.

## 2 Methods

### 2.1 Study Design and Settings

This research utilized a cross‐sectional design based on secondary data from the SHDS 2020. The SHDS is Somalia's first nationally representative, large‐scale demographic and health dataset. The data were collected through a collaborative effort between the Government of Somalia, represented by the Ministry of Planning, Investment, and Economic Development, the Somali National Bureau of Statistics, and the Ministry of Health, in partnership with the United Nations Population Fund (UNFPA). The survey took place between 2018 and 2019.

Somalia consists of 18 pre‐war geographical regions. However, due to security concerns and the presence of al‐Shabaab, data collection for the SHDS was not conducted for two reasons: Lower Shabelle and Middle Jubba. The survey covered the remaining 16 regions, ensuring a broad representation of the Somali population.

### 2.2 Study Population, Data Source, and Sampling Procedure

The source population for this study comprised all pregnant women aged 15–49 years in Somalia. The study population included all pregnant women residing in the selected enumeration areas (EAs), who were surveyed and included in the final analysis. Eligible participants for this study were pregnant women aged 15–49 years who were part of the SHDS 2020 dataset. Women with missing data for the outcome variable and other key predictors were excluded from the analysis to ensure the integrity of the results.

**FIGURE 1:**
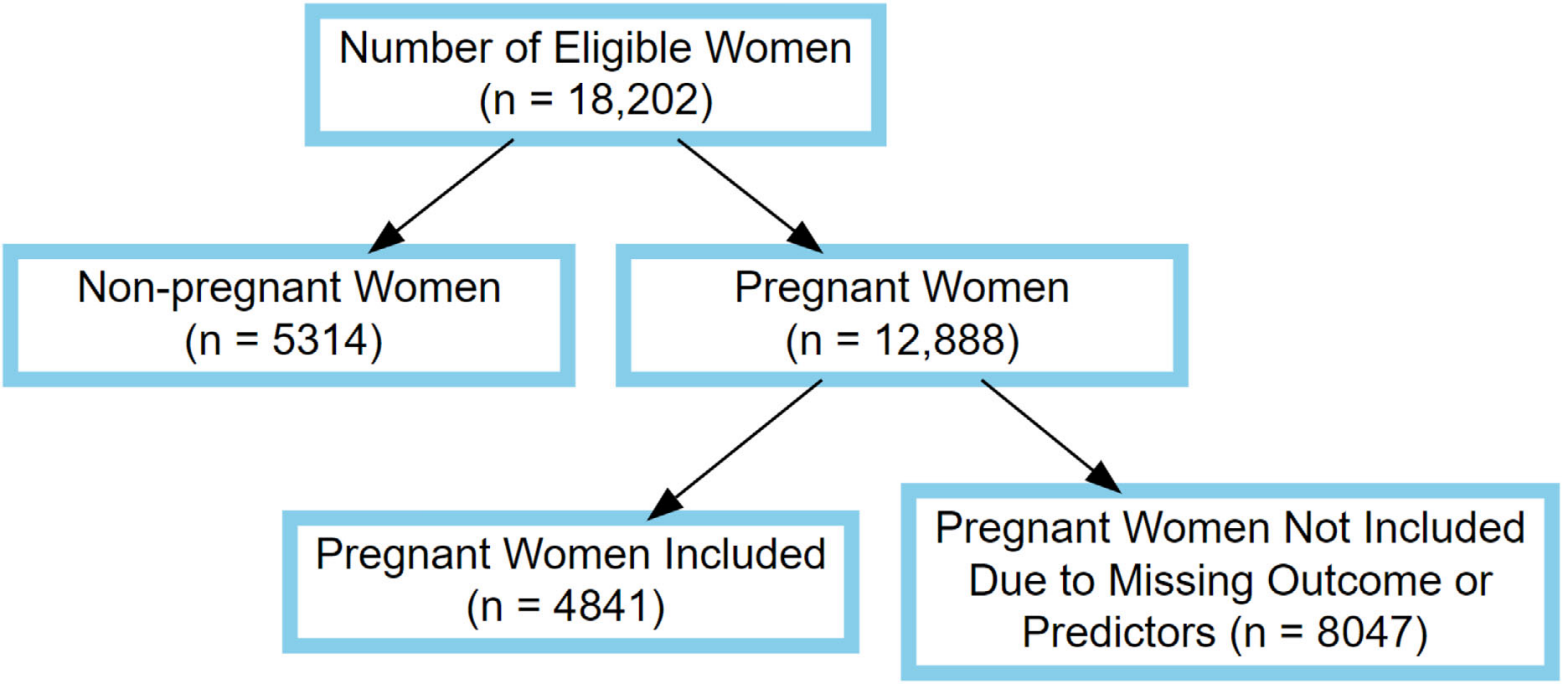
Selection procedure of the final sample of pregnant women included in the study.

The data for this study were obtained from the Somali National Bureau of Statistics (https://microdata.nbs.gov.so/index.php/catalog/50) through the SHDS dataset (file SOIR71SV). The SHDS employed stratified cluster sampling techniques for urban, rural, and nomadic strata to ensure representativeness across different population groups. The survey digitized 10,525 EAs, with 7488 in urban areas and 3037 in rural areas. However, due to security and accessibility constraints, the final sampling frame included 9136 EAs, comprising 7308 urban EAs and 1828 rural EAs. The missing EAs correspond to regions and strata excluded from the survey, such as all strata in Lower Shabelle and Middle Juba regions and the rural and nomadic strata in the Bay region. For urban and rural areas, a three‐stage stratified cluster sampling design was implemented, where primary sampling units (PSUs) and secondary sampling units (SSUs) were selected using probability proportional to size sampling at the first and second stages, and households were systematically sampled at the third stage. For the nomadic population, a two‐stage stratified cluster sampling design was used, where PSUs were selected using probability proportional to size sampling at the first stage, and households were systematically sampled in the second stage. At the household stage, 16,360 households were initially selected for the SHDS sample. Of these, 15,870 households were occupied, and 15,761 households were successfully interviewed, achieving a high response rate (99.3%). After excluding cases with missing data, a final sample of 4841 women aged 15–49 years was included in this analysis. The detailed selection procedure of this final sample is shown in Figure 1.

### 2.3 Study Variables

#### 2.3.1 Dependent Variable

In this study, the dependent variable is the number of TT injections received before giving birth (i.e., 0, 1, 2, …, 8).

#### 2.3.2 Independent Variables

To achieve the study's objective, we selected 25 variables that may influence the number of TT vaccine injections received before birth. These variables include region, residence, maternal age, maternal education, number of ever‐born children, listening to the radio, watching TV, internet usage, and mobile phone ownership. The analysis also considered socioeconomic factors, such as wealth quintile, medical permission from the husband, access to money for treatment, and distance to a health facility. Besides, the reproductive and health‐related factors (e.g., birth order, tetanus injections before pregnancy, number of antenatal care visits, and place of delivery), as well as the decision‐making and household factors (e.g., participation in household decision‐making, husband's age, husband's employment in the last 12 months, woman's current employment status, health‐related decision‐making, and financial decision‐making), were examined. Other factors, such as health insurance coverage and the husband's educational attainment, were also considered.

### 2.4 Data Processing and Data Analysis

Before conducting the analysis, the dataset underwent rigorous cleaning to ensure accuracy, completeness, and consistency. The data cleaning included handling missing values, checking for inconsistencies, and recoding categorical variables where necessary. The data analysis was performed using STATA/MP 17.0, a widely used statistical software for analyzing data.

Frequency distributions were computed to describe the sociodemographic characteristics of the study population. Descriptive statistics and charts were used to calculate summary statistics and visually display the distribution of the TT immunization coverage during pregnancy, providing insights into the data structure and patterns.

As the dependent variable, the number of TT injections before birth, is a count variable (discrete and non‐negative), count regression models were applied to ensure an appropriate estimation framework. Fixed‐effect Poisson regression is used when the count data follows a Poisson distribution, assuming the mean and variance are equal (equidispersion). This model accounts for unobserved heterogeneity by allowing each individual (or unit) to have a fixed intercept, thereby controlling for time‐invariant characteristics. A fixed‐effect negative binomial model is more appropriate if the data exhibit overdispersion (i.e., variance greater than the mean). This model introduces an extra parameter to account for the overdispersion by relaxing the restrictive assumption of equidispersion in the Poisson model. The mixed‐effects Poisson model incorporates both fixed and random effects to account for hierarchical or clustered data structures. This is particularly useful when observations are nested within groups, such as regions and residences in this study. When overdispersion, excessive zeros, and hierarchical structure are present, a mixed‐effects negative binomial (ME‐NB) model is preferable for analysis. It extends the negative binomial model by including random effects, allowing variations across regions and residences while addressing overdispersion and excessive zeros.

To determine the most suitable model, Akaike information criterion (AIC) and Bayesian information criterion (BIC) were used. A lower AIC or BIC value indicates a better model fit. Given that the dataset exhibits both overdispersion and excessive zeros, a negative binomial model is expected to be more appropriate than a Poisson model. Additionally, as the data are structured with two grouping factors—region and residence—an ME‐NB model is preferable as it accommodates hierarchical dependencies, overdispersion, and excessive zeros. Recent research has also demonstrated that multilevel modeling is a robust approach and can benefit from reducing higher order variations [30]. Furthermore, due to the presence of excessive zeros, the non‐use of tetanus injection was modeled separately using a multilevel logistic regression model, allowing for a more accurate representation of the factors influencing non‐utilization.

## 3 Results

A total of 4841 pregnant women were included in this analysis. The sociodemographic characteristics are shown in Table 1. The age distribution of the participants was diverse, with the largest proportion (28.09%) falling within the 25–29 age group, whereas smaller percentages were observed in older age groups, with only 2.31% in the 45–49 range. Geographically, the women were spread across various regions, with the largest groups being Banadir (10.49%) and Woqooyi Galbeed (9.54%). In terms of residence, the majority were urban dwellers (37.51%), followed by nomadic (34.15%) and rural (28.34%) residents. Educationally, a significant majority (86.02%) had no formal education, with only a small proportion completing primary (10.41%) or higher education (0.87%). Media access was limited, as 91.88% of the participants reported never listening to the radio, and 90.29% did not watch TV. Internet access was also low, with 92.15% of participants reporting no internet usage. Mobile phone ownership was widespread, with 80.71% of women owning a mobile phone. Regarding wealth, the distribution was fairly even across the quintiles, with the lowest quintile comprising 26.4% and the highest quintile 14.83%. A majority (53.15%) reported not needing permission from their husbands for medical decisions, and a larger proportion (69.63%) stated they could obtain money for treatment if needed. Access to healthcare was a concern for many, as 65.48% indicated that distance to a health facility was a significant problem. Most women delivered at home (71.89%), and many (68.79%) reported no involvement in household decision‐making. Regarding health decisions, 51% of women reported that their partners made the decisions, whereas 33.24% made joint decisions. Most participants did not have health insurance (99.86%), and most husbands (76.51%) had not attended school. Moreover, the number of antenatal care visits was low, with an average of 1.10 visits, and the average number of ever‐born children was 4.69 (see Table 1).

**TABLE 1:** Sociodemographic characteristics of study participants (*n *= 4841).

Variable name	Categories	Frequency (*n*), mean ± SD	Percentage
**Maternal age**	15–19	283	5.85
	20–24	958	19.79
	25–29	1360	28.09
	30–34	982	20.29
	35–39	825	17.04
	40–44	321	6.63
	45–49	112	2.31
**Region**	Awdal	236	4.88
	Woqooyi Galbeed	462	9.54
	Togdheer	422	8.72
	Sool	393	8.12
	Sanaag	427	8.82
	Bari	134	2.77
	Nugaal	184	3.8
	Mudug	245	5.06
	Galgaduud	242	5
	Hiraan	325	6.71
	Middle Shabelle	242	5
	Banadir	508	10.49
	Bay	137	2.83
	Bakool	258	5.33
	Gedo	280	5.78
	Lower Juba	346	7.15
**Residence**	Urban	1816	37.51
	Rural	1372	28.34
	Nomadic	1653	34.15
**Maternal education**	No Education	4164	86.02
	Primary	504	10.41
	Secondary	131	2.71
	Higher	42	0.87
**Listening to radio**	Not at all	4448	91.88
	At least once a week	269	5.56
	Less than once a week	124	2.56
**Watching TV**	Not at all	4371	90.29
	At least once a week	373	7.71
	Less than once a week	97	2
**Internet usage**	Yes	380	7.85
	No	4461	92.15
**Mobile phone**	Yes	3907	80.71
	No	934	19.29
**Wealth quintile**	Lowest	1278	26.4
	Second	1131	23.36
	Middle	864	17.85
	Fourth	850	17.56
	Highest	718	14.83
**Medical permission from husband**	No	2573	53.15
	Yes	2268	46.85
**Getting money needed for treatment**	No	1470	30.37
	Yes	3371	69.63
**Distance to health facility**	Not big problem	1671	34.52
	Big problem	3170	65.48
**Place delivery**	Home	3480	71.89
	Clinic	1361	28.11
**No participation in decision‐making for house**	No	3330	68.79
	Yes	1320	27.27
	Don't know	191	3.95
**Health decision**	Respondent	747	15.43
	Partner	2469	51
	Joint	1609	33.24
	Other	16	0.33
**Money decision**	Respondent	752	15.53
	Husband	2631	54.35
	Respondent and husband jointly	1441	29.77
	Other	17	0.35
**Tetanus injections before pregnancy**	Yes	1164	24.04
	No	3.559	73.52
	Don't know	118	2.44
**Health insurance**	No	4834	99.86
	Yes	7	0.14
**Husband attended school**	No	3704	76.51
	Yes	955	19.73
	Don't know	182	3.76
**Partner worked in the last 12 months**	Yes	2230	46.06
	No	2575	53.19
	Don't know	36	0.74
**Currently working**	Yes	263	5.43
	No	4578	94.57
**No. of ever‐born children**	—	4.69 ± 2.69	—
**Birth order**	—	4.66 ± 2.64	—
**No. of antenatal care visits**	—	1.10 ± 5.64	—
**Husband age**	—	38.12 ± 14.00	—

**FIGURE 2:**
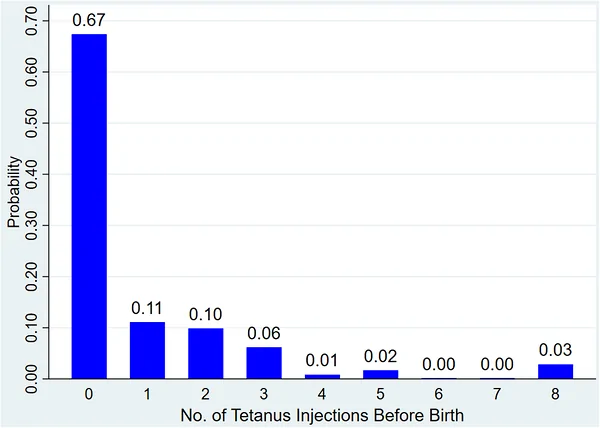
Distribution of the number of TT injections before birth. TT, tetanus toxoid.

To assess the distribution of the antenatal TT doses, we refer to Figure 2, which displays a bar chart illustrating the frequency of each number of TT injections, along with Table 2, which provides summary statistics for these numbers. The majority of women (67.38%) received no tetanus injections, followed by smaller proportions who received one (11.09%), two (9.85%), and three (6.19%) injections. The frequency sharply declines as the number of injections increases beyond three, with only a small percentage of women receiving four or more injections. Table 2 further shows that the mean number of TT injections is approximately 0.48, with a standard deviation of 0.28, indicating some variability in the data. The skewness of 5.40 suggests a strongly right‐skewed distribution, meaning most women received fewer injections, whereas a small number received more. Additionally, the kurtosis value is 32.41 for the distribution with a sharp peak and long tails, particularly due to the low frequencies of women receiving higher TT immunization coverage.

**TABLE 2:** Summary statistics for the number of tetanus toxoid (TT) vaccine injections before birth among pregnant women (*n *= 4841).

Mean	Variance	Skewness	*p* (skewness)	Kurtosis	*p* (kurtosis)
0.348	1.627	5.402	0.000	32.412	0.000

Table 3 compares the tetanus vaccine uptake and selected characteristic between nomadic and non‐nomadic women. Nomadic women had a slightly higher mean number of tetanus vaccine doses than non‐nomadic women (0.38 ± 1.60 vs. 0.33 ± 1.07; *p* < 0.001). Similarly, the proportion of women who received at least two tetanus vaccine doses was significantly higher among nomadic women than among non‐nomadic women (4.17% vs. 1.63%; *p* < 0.001).

**TABLE 3:** Comparison of tetanus vaccine uptake and selected characteristics between nomadic and non‐nomadic women in Somalia (*n *= 4841).

Characteristic	Non‐nomadic (*n *= 3188)	Nomadic (*n *= 1653)	*p* value
**Mean number of tetanus vaccine doses, mean (SD)**	0.33 (1.07)	0.38 (1.60)	<0.001^a^
**Received ≥2 tetanus vaccine doses, *n* (%)**	52 (1.63)	69 (4.17)	<0.001
**Age group, *n* (%)**			0.001
15–19	175 (5.49)	108 (6.53)	
20–24	594 (18.63)	364 (22.02)	
25–29	886 (27.79)	474 (28.68)	
30–34	700 (21.96)	282 (17.06)	
35–39	551 (17.28)	274 (16.58)	
40–44	210 (6.59)	111 (6.72)	
45–49	72 (2.26)	40 (2.42)	
**Education, *n* (%)**			<0.001
No education	2537 (79.58)	1627 (98.43)	
Primary	479 (15.03)	25 (1.51)	
Secondary	131 (4.11)	0 (0.00)	
Higher	41 (1.29)	1 (0.06)	
**Wealth quintile, *n* (%)**			<0.001
Lowest	300 (9.41)	978 (59.17)	
Second	553 (17.35)	578 (34.97)	
Middle	827 (25.94)	37 (2.24)	
Fourth	820 (25.72)	30 (1.81)	
Highest	688 (21.58)	30 (1.81)	
**Radio exposure, *n* (%)**			<0.001
Not at all	2835 (88.93)	1613 (97.58)	
At least once/week	245 (7.69)	24 (1.45)	
Less than once/week	108 (3.39)	16 (0.97)	
**Television exposure, *n* (%)**			<0.001
Not at all	2730 (85.63)	1641 (99.27)	
At least once/week	368 (11.54)	5 (0.30)	
Less than once/week	90 (2.82)	7 (0.42)	
**Place of delivery, *n* (%)**			<0.001
Home	2018 (63.30)	1462 (88.45)	
Health facility	1170 (36.70)	191 (11.55)	
**Health insurance, *n* (%)**			0.267
No	3182 (99.81)	1652 (99.94)	
Yes	6 (0.19)	1 (0.06)	

*Note:* Values are presented as *n* (%) unless otherwise indicated.

^a^ *p* value for the mean number of tetanus vaccine doses was obtained using the Wilcoxon rank‐sum (Mann–Whitney) test because the outcome was highly skewed. *p* values for categorical variables were obtained using Pearson's *χ* ^2^ test.

Significant differences were also observed in the sociodemographic characteristics of the two populations. Compared with non‐nomadic women, nomadic women were substantially more likely to have no formal education (98.4% vs. 79.6%), belong to the lowest wealth quintile (59.2% vs. 9.4%), have no exposure to radio (97.6% vs. 88.9%) or television (99.3% vs. 85.6%), and deliver at home (88.5% vs. 63.3%) (all *p* < 0.001). In contrast, health insurance coverage was extremely low in both groups and did not differ significantly between nomadic and non‐nomadic women (*p* = 0.267).

To assess the multilevel factors associated with the number of TT injections before birth, the distribution of the outcome variable was first assessed. As shown in Table 2, the variance was approximately five times greater than the mean, indicating substantial overdispersion and suggesting that the standard Poisson model was unlikely to provide an adequate fit. Additionally, the presence of excessive zeros in the data further complicates modeling, as the Poisson model assumes that zeros follow the same distribution as positive counts. In such cases, a negative binomial model is more appropriate because it accounts for both overdispersion and an excess of zeros. Moreover, the hierarchical structure of the data must be considered, so we compared fixed‐ and mixed‐effects models for both Poisson and negative binomial distributions using model selection criteria. Table 4 shows the model selection results, where the ME‐NB model (AIC = 6191.847, BIC = 6509.696) performs best compared to other models, indicating it is the most suitable choice for these data.

**TABLE 4:** Model selection criteria.

Model	AIC	BIC
Fixed‐effects poisson	7804.87	8200.384
Fixed‐effects negative binomial	6196.177	6598.239
Mixed‐effects poisson	7702.568	7799.841
Mixed‐effects negative binomial	**6191.847**	**6509.696**

Abbreviations: AIC, Akaike information criterion; BIC, Bayesian information criterion.

Bold values represent the lowest information criteria.

Table 5 presents the results from the ME‐NB regression modeling. Statistically significant factors include age, wealth quintile, seeking medical permission from the husband, tetanus injections before pregnancy, number of antenatal care visits, currently working status, place of delivery, and having health insurance. Specifically, women aged 20–24 years had 1.62 times higher rates of tetanus injections (IRR = 1.624, 95% CI = 1.092–2.417) compared to those aged 15–19 years. Women in the highest wealth quintile had 1.49 times higher rates (IRR = 1.487, 95% CI = 1.014–2.180) than those in the lowest wealth quintile. Women who usually await or seek medical permission from their husbands had lower rates of tetanus injections (IRR = 0.761, 95% CI = 0.619–0.935). Women who had received tetanus injections before pregnancy had 1.76 times higher rates (IRR = 1.764, 95% CI = 1.44–2.161). Additionally, for every additional antenatal care visit, women had 1.02 times higher rates of tetanus injections (IRR = 1.024, 95% CI = 1.006–1.041), and women who were currently working had 1.41 times higher rates (IRR = 1.406, 95% CI = 1.003–1.970). Women who delivered in a clinic had 1.23 times higher rates of tetanus injections than those who delivered elsewhere (IRR = 1.227, 95% CI = 1.006–1.041). Moreover, having health insurance was associated with a significantly higher rate of tetanus injections, as women with health insurance had 5.24 times higher rates (IRR = 5.242, 95% CI = 1.038–26.471) compared to those without insurance.

**TABLE 5:** Multilevel factors associated with the number of tetanus‐toxoid (TT) vaccine injections before birth among pregnant women in Somalia (*n *= 4841).

Variable	Categories	IRR	Std. err.	*z*	*p *> |*z*|	95% CI lower	95% CI upper
**Residence**	Rural	Ref					
	Urban	1.013	0.171	0.08	0.938	0.728	1.41
	Nomadic	1.389	0.251	1.82	0.069	0.975	1.979
**Maternal age**	15–19	Ref					
	20–24	1.624	0.329	2.39	0.017	1.092	2.417
	25–29	1.234	0.255	1.02	0.308	0.823	1.851
	30–34	0.986	0.227	−0.06	0.95	0.627	1.547
	35–39	1.572	0.381	1.86	0.062	0.977	2.529
	40–44	1.813	0.51	2.11	0.034	1.044	3.148
	45–49	2.089	0.75	2.05	0.04	1.033	4.222
**Maternal education**	No formal education	Ref					
	Primary	1.143	0.167	0.91	0.36	0.858	1.523
	Secondary	1.014	0.275	0.05	0.959	0.596	1.725
	Higher	0.702	0.313	−0.79	0.427	0.293	1.681
**No. of ever‐born children**	—	1.074	0.075	1.02	0.31	0.935	1.232
**Listening to radio**	Not at all	Ref					
	At least once a week	0.999	0.185	−0.01	0.994	0.694	1.435
	Less than once a week	1.603	0.404	1.87	0.061	0.977	2.628
**Watching TV**	Not at all	Ref					
	At least once a week	0.895	0.162	−0.61	0.54	0.628	1.276
	Less than once a week	0.83	0.25	−0.62	0.536	0.459	1.498
**Internet usage**	No	Ref					
	Yes	1.007	0.18	0.04	0.97	0.709	1.428
**Mobile phone**	No	Ref					
	Yes	1.064	0.125	0.52	0.6	0.844	1.339
**Wealth quintile**	Lowest	Ref					
	Second	0.87	0.12	−1.01	0.313	0.664	1.14
	Middle	1.235	0.211	1.24	0.216	0.883	1.727
	Fourth	1.214	0.217	1.09	0.277	0.855	1.723
	Highest	1.487	0.29	2.03	0.042	1.014	2.18
**Medical permission from husband**	No	Ref					
	Yes	0.761	0.08	−2.6	0.009	0.619	0.935
**Getting money needed for treatment**	No	Ref					
	Yes	1.038	0.14	0.28	0.781	0.797	1.352
**Distance to health facility**	Not big problem	Ref					
	Big problem	1.08	0.142	0.58	0.559	0.834	1.398
**Birth order**	—	0.905	0.067	−1.36	0.173	0.783	1.045
**Tetanus injections before pregnancy**	No	Ref					
	Yes	1.764	0.182	5.49	0.000	1.440	2.161
	Don't know	15.655	3.124	13.78	0.000	10.587	23.148
**No. of antenatal care visits**	—	1.024	0.009	2.75	0.006	1.006	1.041
**Place delivery**	Home	Ref					
	Clinic	1.227	0.124	2.03	0.043	1.006	1.041
**No participation in decision‐making for house**	No	Ref					
	Yes	1.016	0.100	0.16	0.874	1.006	1.041
	Don't know	1.567	0.312	2.26	0.024	0.836	1.233
**Husband age**	—	0.994	0.003	−1.81	0.070	0.987	1.000
**Partner worked in the last 12 months**	No	Ref					
	Yes	0.957	0.095	−0.44	0.657	0.788	1.162
	Don't know	1.535	0.661	1.0	0.32	0.66	3.572
**Currently working**	No	Ref					
	Yes	1.406	0.242	1.98	0.047	1.003	1.97
**Health decision**	Respondent	Ref					
	Partner	1.175	0.203	0.94	0.35	0.837	1.648
	Joint	1.008	0.188	0.04	0.966	0.698	1.454
	Other	0.626	0.501	−0.59	0.558	0.13	3.008
**Money decision**	Respondent	Ref					
	Husband	0.783	0.13	−1.47	0.142	0.564	1.085
	Respondent and husband jointly	0.843	0.159	−0.91	0.363	0.582	1.219
	Other	0.933	0.682	−0.09	0.924	0.222	3.906
**Health insurance**	No	Ref					
	Yes	5.242	4.331	2.01	0.045	1.038	26.471
**Husband attended school**	No	Ref					
	Don't know	0.931	0.213	−0.31	0.757	0.594	1.459
	Yes	1.175	0.142	1.34	0.180	0.927	1.489
**Constant**	—	0.163	0.050	−5.90	0.000	0.089	0.297
**lnalpha**	—	1.307	0.066			1.177	1.436
**Region**	var(constant)	0.003	0.032			0.000	261,862
**Region > residence**	var(constant)	0.101	0.050			0.038	0.265

As these data have excessive zeros, we modeled the multilevel factors associated with zero tetanus vaccine injection (the zero‐component) before birth among pregnant women in Somalia using a multilevel binary logistic regression model. The results are shown in Table 6. The findings indicate several significant factors. Women residing in nomadic areas were 1.40 times more likely to have no tetanus vaccine injection compared to those in rural areas (AOR = 1.397, 95% CI = 1.056–1.849, *p* = 0.019). Women aged 30–34 were significantly more likely to have no vaccine, with 1.56 times higher odds than those aged 15–19 (AOR = 1.564, 95% CI = 1.018–2.402, *p* = 0.041). The number of antenatal care visits was also a key factor, with each additional visit decreasing the odds of no vaccine by 2% (AOR = 0.981, 95% CI = 0.970–0.992, *p* = 0.001). Women who had previously received a tetanus vaccine before pregnancy were less likely to have no vaccine during pregnancy (AOR = 0.275, 95% CI = 0.229–0.330, *p* < 0.001). Additionally, the wealth quintile was significant, with women in the highest wealth quintile having 34% lower odds of no vaccine than those in the lowest quintile (AOR = 0.663, 95% CI = 0.453–0.970, *p* = 0.034). Women who delivered at the clinic were also less likely to have no vaccine (AOR = 0.702, 95% CI = 0.583–0.846, *p* < 0.001). Mobile phone ownership was associated with a reduced likelihood of having no vaccine (AOR = 0.765, 95% CI = 0.595–0.985, *p* = 0.037). Lastly, women whose husbands attended school had 21% lower odds of no vaccine compared to those whose husbands did not attend school (AOR = 0.789, 95% CI = 0.636–0.978, *p* = 0.031).

**TABLE 6:** Multilevel factors associated with zero tetanus vaccine injections before birth among pregnant women in Somalia (*n* = 4841).

Variable	Categories	AOR	Std. err.	*z*	*p *> |*z*|	95% CI lower	95% CI upper
**Residence**	Rural	Ref					
	Urban	1.015	0.114	0.130	0.895	0.814	1.266
	Nomadic	1.397	0.200	2.340	0.019	1.056	1.849
**Maternal age**	15–19	Ref					
	20–24	0.945	0.179	−0.300	0.767	0.652	1.371
	25–29	1.187	0.230	0.890	0.375	0.813	1.735
	30–34	1.564	0.343	2.040	0.041	1.018	2.402
	35–39	1.271	0.297	1.030	0.305	0.804	2.011
	40–44	1.059	0.297	0.200	0.838	0.611	1.835
	45–49	1.396	0.536	0.870	0.385	0.657	2.964
**Maternal education**	No formal education	Ref					
	Primary	0.979	0.128	−0.160	0.873	0.759	1.264
	Secondary	1.138	0.274	0.540	0.590	0.711	1.823
	Higher	1.095	0.422	0.240	0.813	0.515	2.329
**No. of ever‐born children**	—	0.979	0.081	−0.260	0.798	0.833	1.150
**Listening to radio**	Not at all	Ref					
	At least once a week	0.889	0.146	−0.720	0.471	0.645	1.225
	Less than once a week	0.567	0.130	−2.480	0.013	0.362	0.888
**Watching TV**	Not at all	Ref					
	At least once a week	0.983	0.155	−0.110	0.914	0.721	1.340
	Less than once a week	1.518	0.430	1.480	0.140	0.872	2.644
**Internet usage**	No	Ref					
	Yes	1.085	0.175	0.510	0.613	0.791	1.487
**Mobile phone**	No	Ref					
	Yes	0.765	0.098	−2.080	0.037	0.595	0.985
**Wealth quintile**	Lowest	Ref					
	Second	1.008	0.152	0.060	0.955	0.751	1.354
	Middle	0.869	0.150	−0.810	0.417	0.620	1.219
	Fourth	0.682	0.120	−2.170	0.030	0.483	0.964
	Highest	0.663	0.129	−2.120	0.034	0.453	0.970
**Medical permission from husband**	No	Ref					
	Yes	1.077	0.112	0.720	0.474	0.879	1.319
**Getting money needed for treatment**	No	Ref					
	Yes	0.982	0.127	−0.140	0.891	0.762	1.267
**Distance to health facility**	Not big problem	Ref					
	Big problem	0.920	0.117	−0.660	0.512	0.718	1.180
**Birth order**	—	1.082	0.092	0.930	0.351	0.917	1.277
**Tetanus injections before pregnancy**	No	Ref					
	Yes	0.275	0.025	−13.970	0.000	0.229	0.330
	Don't know	0.115	0.023	−10.630	0.000	0.077	0.171
**No. of antenatal care visits**	—	0.981	0.006	−3.370	0.001	0.970	0.992
**Place delivery**	Home	Ref					
	Clinic	0.702	0.067	−3.730	0.000	0.583	0.846
**No participation in decision‐making for house**	No	Ref					
	Yes	0.929	0.090	−0.760	0.444	0.769	1.122
	Don't know	0.883	0.193	−0.570	0.569	0.575	1.356
**Husband age**	—	1.001	0.003	0.170	0.863	0.994	1.007
**Partner worked in the last 12 months**	No	Ref					
	Yes	0.868	0.084	−1.470	0.142	0.718	1.049
	Don't know	0.445	0.184	−1.960	0.050	0.198	1.002
**Currently working**	No	Ref					
	Yes	0.918	0.156	−0.500	0.616	0.658	1.281
**Health decision**	Respondent	Ref					
	Partner	0.922	0.153	−0.490	0.624	0.666	1.276
	Joint	0.944	0.169	−0.320	0.748	0.666	1.340
	Other	1.550	1.231	0.550	0.581	0.327	7.350
**Money decision**	Respondent	Ref					
	Husband	1.113	0.181	0.660	0.509	0.810	1.530
	Respondent and husband jointly	0.920	0.167	−0.460	0.645	0.645	1.312
	Other	0.606	0.417	−0.730	0.467	0.157	2.335
**Health insurance**	No	Ref					
	Yes	0.189	0.167	−1.880	0.060	0.033	1.073
**Husband attended school**	No	Ref					
	Don't know	1.008	0.205	0.040	0.967	0.676	1.503
	Yes	0.789	0.087	−2.160	0.031	0.636	0.978
**Constant**	—	10.157	3.037	7.750	0.000	5.652	18.251
**Region**	var(constant)	0.037	0.026			0.009	0.148

## 4 Discussion

The present study employed an ME‐NB model to examine the multilevel factors associated with the number of TT vaccine injections before birth among pregnant women in Somalia. The findings reveal a concerning pattern of low tetanus vaccine coverage, with 67.38% of women receiving no injections. This highlights a critical gap in maternal immunization efforts, which is in line with previous research indicating that low‐income and conflict‐affected regions often struggle with vaccine coverage [31, 32]. The low maternal tetanus vaccination coverage in Somalia is unlikely to be explained solely by vaccine availability. Somalia does not have domestic vaccine manufacturing capacity and relies primarily on international procurement mechanisms and global vaccine supply systems [22]. Vaccine procurement and distribution are supported through collaborations involving the Federal Ministry of Health, the WHO, UNICEF, Gavi, and other development partners [22]. Although international partners have played a major role in ensuring vaccine availability, challenges remain in delivering vaccines effectively to all populations. Supply chain limitations, cold‐chain requirements, insecurity, geographic barriers, and weak healthcare infrastructure may affect timely vaccine delivery, particularly among rural and nomadic communities. Therefore, improving TT vaccination coverage requires strengthening not only vaccine supply but also last‐mile delivery systems, community outreach programs, and integration of vaccination services into antenatal care.

Although research demonstrated that vaccination is very important to maternal health and robust child‐rearing practices, this somehow depends on household economic status and awareness [33]. The strong right‐skewed distribution of the TT doses suggests that although a small fraction of women receive multiple doses, the majority remain unprotected. This distribution pattern aligns with studies in other low‐resource settings where healthcare access disparities lead to highly uneven immunization uptake [34].

Our model selection process demonstrated that an ME‐NB model was the most suitable for analyzing these data, as it accounts for overdispersion and the excess of zero counts. The variance‐to‐mean ratio was approximately five, reinforcing the presence of overdispersion. Additionally, the AIC and BIC confirmed the superior fit of the ME‐NB model over traditional Poisson models. This methodological choice is consistent with previous studies on excess zero observations, overdispersion, and count data [35, 36].

Among individual‐level factors, maternal age was significantly associated with the number of TT injections received. Women aged 20–24 had a 1.62 times higher rate of vaccination than those aged 15–19. This may be due to increased health awareness and better healthcare‐seeking behavior among older pregnant women. Similar findings have been reported in studies on maternal health services in sub‐Saharan Africa, where younger women often face barriers, such as limited autonomy and health literacy [37, 38, 39]. Additionally, women who had received tetanus injections before pregnancy had 1.76 times higher vaccination rates before birth, suggesting that prior exposure to immunization programs fosters continued participation in maternal health services. Studies indicate that women previously receiving vaccines had significantly higher uptake rates, suggesting that promoting vaccine use and addressing hesitancy can improve tetanus injection uptake rates [40]. Adverse events following immunization (AEFI) are an important consideration in maintaining public confidence in vaccination programs. Although mild reactions following tetanus‐containing vaccines, such as temporary pain at the injection site or fever, are commonly reported, serious adverse events are rare [41]. Effective AEFI surveillance systems are essential to rapidly identify, investigate, and communicate vaccine safety information to communities. In Somalia, available evidence does not indicate that reported AEFI is a major documented cause of low maternal tetanus vaccination coverage. Instead, vaccine hesitancy is more likely influenced by broader factors, including limited awareness, misinformation, inadequate counseling during pregnancy, accessibility barriers, and reduced interaction with health services [42]. Strengthening vaccine safety communication, training healthcare workers on AEFI reporting, and improving community trust may contribute to increased acceptance of maternal immunization.

Socioeconomic factors also played a crucial role in tetanus vaccine uptake. Women in the highest wealth quintile had 1.49 times higher rates of tetanus injections compared to those in the lowest quintile. This finding is consistent with global studies highlighting the positive correlation between household wealth and healthcare utilization [3, 43, 44]. Financial constraints may hinder access to health facilities, preventing women from receiving adequate prenatal care and vaccinations. Furthermore, women who were currently employed had 1.41 times higher vaccination rates, possibly due to increased financial independence and decision‐making power in seeking healthcare services. Employment provides women with the resources to afford healthcare and the autonomy to prioritize vaccination, reducing reliance on family members for health‐related decisions. Additionally, workplaces may serve as sources of health information, raising awareness about the importance of vaccinations. Studies have shown a positive correlation between employment and tetanus vaccine uptake, reinforcing the idea that financial stability and empowerment contribute to better health‐seeking behavior [3, 45, 46, 47]. This suggests that promoting economic opportunities for women could indirectly improve vaccination rates by enhancing their access to healthcare services and their ability to make independent health decisions.

Social and cultural factors also influenced tetanus vaccination rates. Women who required medical permission from their husbands had significantly lower vaccination rates (IRR = 0.761), underscoring the impact of gender dynamics on maternal health decisions. Similar barriers have been documented in patriarchal societies where women's healthcare choices are often dictated by their spouses [48]. Studies have shown that women's decision‐making autonomy significantly impacts their likelihood of receiving tetanus vaccinations during pregnancy [15]. Therefore, interventions promoting gender equality in healthcare decision‐making could help bridge this gap and enhance vaccine uptake among pregnant women.

Healthcare access indicators, such as the number of ANC visits, were strongly associated with tetanus vaccine uptake. For every additional ANC visit, the rate of tetanus injections increased by 1.024 times. This aligns with findings from previous studies, where frequent ANC visits were linked to higher immunization rates [10, 49, 50, 51, 52]. ANC visits provide a critical opportunity for healthcare providers to educate pregnant women about the importance of tetanus vaccination and ensure its administration. Moreover, women who delivered in clinics had higher vaccination rates, reinforcing the importance of institutional healthcare settings in promoting immunization adherence. Previous studies have shown that institutional delivery significantly influences neonatal tetanus protection [15, 53]. Research suggests that institutional deliveries provide better healthcare services for mothers and facilitate access to essential vaccinations, highlighting the importance of improving healthcare facility delivery rates to enhance maternal and neonatal health outcomes [54].

Health insurance coverage was another significant determinant of tetanus vaccine uptake. Women with health insurance had 5.24 times higher rates of tetanus injections compared to those without insurance. This dramatic difference highlights the role of financial barriers in vaccine accessibility, emphasizing the need for policies that expand insurance coverage and reduce out‐of‐pocket expenses for maternal healthcare services. These findings resonate with previous research indicating that insured women are more likely to utilize preventive health services, including vaccinations [34].

In addition to assessing the TT doses received, we also modeled the likelihood of receiving zero injections using a multilevel binary logistic regression model. Women residing in nomadic areas were 1.40 times more likely to have no tetanus injection than those in rural areas. The mobility of nomadic populations poses a challenge for routine healthcare delivery, necessitating targeted outreach programs to improve vaccine accessibility in these communities. Additionally, women aged 30–34 were significantly more likely to remain unvaccinated compared to those aged 15–19, possibly due to misconceptions about the necessity of tetanus immunization in later pregnancies, as observed in prior studies on maternal vaccine hesitancy [55].

Women who had previously received a tetanus vaccine before pregnancy were significantly less likely to have no vaccine during pregnancy. This suggests that prior exposure to tetanus immunization fosters a continued inclination toward maternal vaccinations, possibly due to increased awareness and trust in healthcare services. Public health interventions should focus on reinforcing these patterns by encouraging vaccination during adolescence and early reproductive years. Additionally, integrating maternal immunization reminders into routine healthcare visits could further improve coverage rates.

The wealth quintile was another vital determinant, with women in the highest wealth quintile having 34% lower odds of receiving no vaccine compared to those in the lowest quintile. This reinforces the well‐established link between economic status and healthcare access, where financial stability enables better utilization of preventive health measures. Additionally, mobile phone ownership was associated with a reduced likelihood of having no vaccine. This aligns with a study that emphasized the potential of mobile health (mHealth) interventions in improving maternal immunization [56]. Outreach programs leveraging mobile technology for vaccine reminders and education could significantly enhance immunization uptake. Thus, it could be a valuable tool for increasing maternal vaccine coverage in Somalia and other resource‐limited settings. Moreover, a collaborative community healthcare with smart technology‐based system [57] might be useful to provide timely affordable healthcare and vaccination services in many countries [58].

The number of ANC visits remained a significant protective factor against receiving zero tetanus injections, with each additional visit reducing the odds by 2%. This underscores the importance of integrating vaccination services into routine ANC visits to increase coverage rates. Regular ANC visits offer key opportunities for healthcare providers to educate pregnant women, address concerns, and ensure timely vaccination. In addition, delivering at a clinic or health facility further protects against zero tetanus injections, as these settings often have better access to vaccines and trained personnel. Combining increased ANC attendance with facility‐based delivery can significantly improve maternal immunization rates, ensuring better health outcomes for mothers and infants.

An important factor associated with a lower likelihood of receiving zero tetanus injections is the husband's educational level. Studies have shown that educated husbands are more likely to encourage their wives to receive vaccinations, including TT injections [59]. These findings suggest that improving the education of both mothers and their husbands could enhance vaccination uptake, as educated husbands are more likely to support and encourage their wives to participate in preventive health measures like vaccination. Thus, strategies to increase male involvement in maternal healthcare decisions could be pivotal in improving immunization coverage and maternal and child health outcomes.

Apart from these factors, Somalia has implemented several strategies to strengthen immunization coverage through collaboration between governmental institutions and international organizations. The national immunization program emphasizes strengthening routine immunization services, improving vaccine access, conducting supplementary immunization activities, enhancing disease surveillance, and integrating vaccination with maternal and child health services [22]. International agencies, including WHO, UNICEF, Gavi, and other humanitarian and development partners, support Somalia through vaccine procurement, technical assistance, health worker training, cold‐chain strengthening, monitoring and evaluation, and community mobilization activities [22]. These partnerships are particularly important in fragile settings where health‐system limitations affect routine healthcare delivery. On the basis of the findings of this study, strategies to improve TT vaccination coverage should prioritize integration of tetanus vaccination into every ANC contact, expansion of outreach services for nomadic populations, strengthening community health worker engagement, improving male involvement in maternal healthcare decisions, and utilizing mHealth technologies for vaccine reminders and education. These approaches should be implemented alongside broader health system strengthening efforts to achieve sustainable MNTE.

Despite the strengths of this study, some limitations must be acknowledged. First, the data were cross‐sectional, limiting causal inferences between the identified factors and tetanus vaccine uptake. Longitudinal studies would provide stronger evidence on the determinants of maternal immunization. Second, self‐reported vaccination status may be subject to recall bias, potentially affecting the accuracy of the reported number of TT injections. Third, although the ME‐NB model accounted for individual‐ and community‐level factors, unmeasured confounders, such as cultural beliefs and vaccine hesitancy, may still influence the results. Lastly, the study was conducted in Somalia, and the findings may not be generalizable to other settings with different healthcare infrastructure and sociocultural dynamics. Future research should explore qualitative aspects of vaccine hesitancy and assess the impact of targeted interventions to improve tetanus immunization among pregnant women.

## 5 Conclusion

This study aimed to examine the multilevel factors associated with the number of TT vaccine injections before birth among pregnant women in Somalia using an ME‐NB model. The findings revealed that 67.38% of women received no tetanus injections, highlighting a significant gap in maternal immunization. Factors positively associated with the number of TT injections included maternal age, with older women more likely to receive vaccinations than younger women, not requiring spousal permission for medical decisions, prior tetanus immunization, employment status, higher household wealth, health insurance coverage, ANC visits, and institutional deliveries. In contrast, factors associated with a higher likelihood of receiving zero tetanus injections included residing in nomadic areas, older maternal age (30–34 years), lower socioeconomic status, uneducated spouse, no prior tetanus immunization, reduced number of ANC visits, not having a mobile phone, and deliveries at home. Given these findings, efforts to improve tetanus immunization should focus on increasing ANC attendance, integrating vaccination into routine maternal healthcare, addressing socioeconomic disparities, and leveraging mHealth technologies to enhance vaccine awareness and accessibility, particularly among underserved and nomadic populations. Policymakers should also implement strategies that promote women's autonomy in healthcare decision‐making and expand financial support mechanisms, such as health insurance, to ensure equitable vaccine access. Addressing these barriers through targeted interventions will be crucial in improving maternal and neonatal health outcomes in Somalia.

## Author Contributions

**Jama Mohamed** prepared the original draft and did the formal analysis and conceptualization. **Hamse Mohamed Amoud** wrote the introduction and the methodology. **Ahmed Ismail Mohamed** participated in the methodology and data curation. **Abdisalam Hassan Muse** participated in the review, editing, and correcting throughout the manuscript. **Azizur Rahman** participated in the review, editing, correcting, and supervising the work. **Saralees Nadarajah** participated in the review, editing, and supervising the work. All authors agreed and approved the final version of the manuscript.

## Funding

The authors have nothing to report.

## Ethics Statement

The authors have nothing to report.

## Conflicts of Interest

The authors declare no conflicts of interest.

## Data Availability

Data from this study were sourced from the Somali National Bureau of Statistics, available at https://microdata.nbs.gov.so/index.php/catalog/50.
